# A rare case of resection of a mucinous cystic neoplasm originating from the extrahepatic bile duct with cholangioscopic imaging

**DOI:** 10.1002/deo2.349

**Published:** 2024-03-15

**Authors:** Yoshiharu Masaki, Yujiro Kawakami, Keisuke Ishigami, Ayako Murota, Masahiro Shitani, Kazuharu Kukita, Yasutoshi Kimura, Keiko Segawa, Tadashi Hasegawa, Hiroshi Nakase

**Affiliations:** ^1^ Department of Gastroenterology and Hepatology Sapporo Medical University School of Medicine Hokkaido Japan; ^2^ Department of Gastroenterology and Hepatology JR Sapporo Hospital Hokkaido Japan; ^3^ Department of Surgery Surgical Oncology and Science Sapporo Medical University School of Medicine Hokkaido Japan; ^4^ Department of Surgical Pathology Sapporo Medical University School of Medicine Hokkaido Japan; ^5^ Department of Pathology Kushiro City General Hospital Hokkaido Japan

**Keywords:** biliary cystadenoma, cholangioscopic imaging, extrahepatic bile duct, mucinous cystic neoplasm, obstructive jaundice

## Abstract

A 29‐year‐old woman was admitted to our hospital for examination of obstructive jaundice and an extrahepatic bile duct lesion. Contrast‐enhanced computed tomography revealed a 20 mm cystic lesion with a thin external capsule in the common hepatic duct. Cholangioscopy revealed translucent oval masses with capillary vessels attached to the bile duct walls. The surface was mostly smooth yet partially irregular with redness, suggesting that the masses were epithelial neoplasms. Histological findings of cholangioscopy‐guided targeted biopsies of the mass showed subepithelial spindle cell proliferation with no atypical epithelium. The patient underwent an extrahepatic bile duct resection to confirm the pathological diagnosis. Immunohistochemistry of surgical specimens revealed that the spindle cells were positive for estrogen and progesterone receptors. Finally, the cystic lesion with ovarian‐like stroma was diagnosed as a mucinous cystic neoplasm with low‐grade intraepithelial neoplasia. This is the first report of cholangioscopic imaging of a biliary mucinous cyctic neoplasm. Cholangioscopic imaging can be helpful in the differential diagnosis of biliary neoplasms and in the determination of treatment strategies.

## INTRODUCTION

Mucinous cystic neoplasms (MCNs) are rare variants of cystic tumors that often arise from the pancreas and hepatobiliary tract.^1^ However, MCNs originating from the extrahepatic bile duct are extremely rare, and their imaging findings are not well known. Peroral digital cholangioscopy has recently been developed and is performed for the differential diagnosis of biliary strictures and horizontal tumor extension of cholangiocarcinoma.^2^ Here, we report a case of a resected MCN originating from the extrahepatic bile duct, for which we could observe the details using digital cholangioscopy.

## CASE REPORT

A 29‐year‐old woman was admitted to our hospital for examination of obstructive jaundice and an extrahepatic bile duct lesion. The patient was asymptomatic but had type 2 diabetes mellitus and bronchial asthma. Laboratory data showed elevated total bilirubin levels (8.2 mg/dL) and transaminase levels (aspartate transaminase: 67 U/L, alanine transaminase: 32 U/L). The serum carbohydrate antigen CA19‐9 level was not elevated (18.5 U/mL).

**FIGURE 1:**
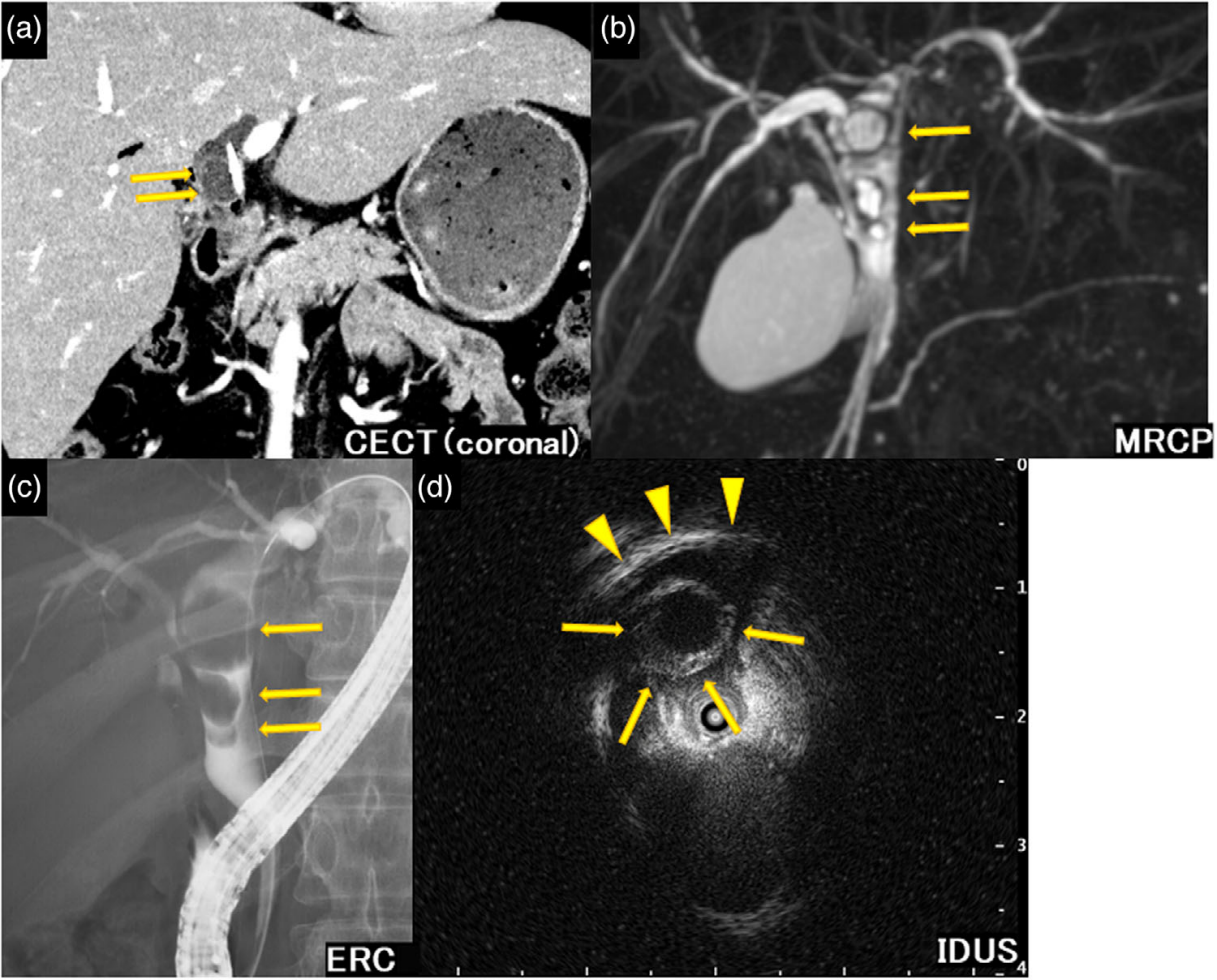
Radiological findings of the extrahepatic bile duct lesion. (a) Contrast‐enhanced computed tomography (CECT) revealed a 20 mm cystic lesion with a thin external capsule in the common hepatic duct. (b) Magnetic resonance cholangiopancreatography (MRCP) showed strong hyperintensity in the extrahepatic bile duct on T2 weighted imaging, suggesting that the lesion mainly consisted of a cystic component. (c) Endoscopic retrograde cholangiography (ERC) showing diffuse bile duct dilation and three round contrast defects in the extrahepatic bile duct. (d) Intraductal ultrasonography (IDUS) shows anechoic lesions with a 2–5 mm low echoic external capsule (arrow); however, the bile duct wall is not thickened (arrowhead).

Contrast‐enhanced computed tomography revealed a 20 mm cystic lesion with a thin external capsule in the common hepatic duct (Figure 1a). Magnetic resonance cholangiopancreatography showed strong hyperintensity in the extrahepatic bile duct on T2 weighted imaging, suggesting that the lesion consisted mainly of a cystic component (Figure 1b). Endoscopic retrograde cholangiography (ERC) revealed diffuse bile duct dilation and three round‐shaped contrast defects in the extrahepatic bile duct (Figure 1c). Intraductal ultrasonography showed anechoic lesions with 2–5 mm low echoic external capsules; however, the bile duct wall was not thickened (Figure 1d).

**FIGURE 2:**
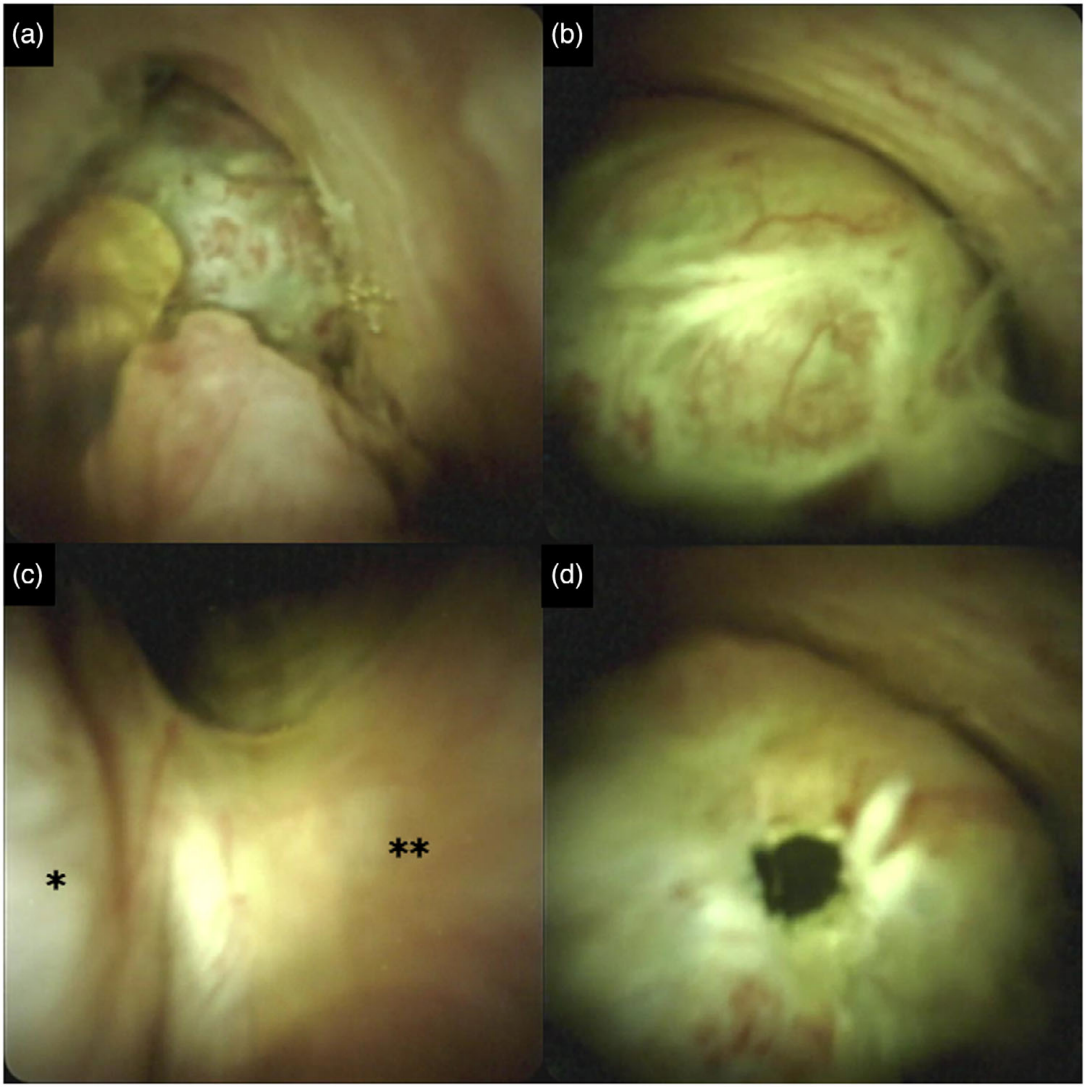
Image findings of peroral digital cholangioscopy (SpyScope DS II; Boston Scientific) for the extrahepatic bile duct mass. (a) Cholangioscopic imaging revealed translucent oval‐shaped masses with capillary vessels. The surface was mostly smooth yet partially irregular with redness. (b) Cholangioscopic imaging in proximity. (c) The mass is attached to the bile duct wall (* mass; ** bile duct wall). (d) After targeted biopsy using dedicated biopsy forceps (SpyBite Max; Boston Scientific), the lesion had a small hole.

**FIGURE 3:**
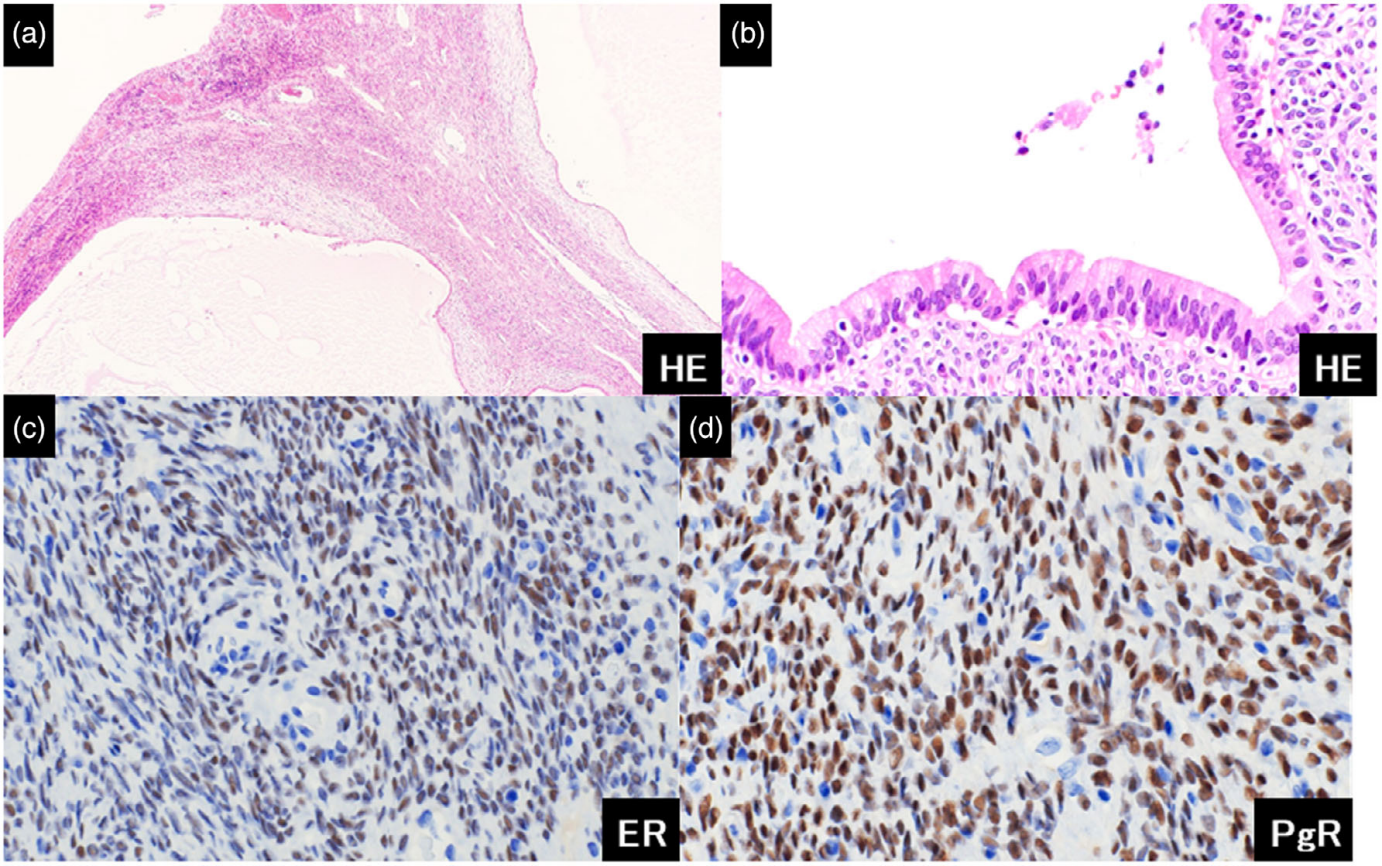
Histopathological findings of the resected specimen. (a, b) Histopathological evaluation demonstrating a layer of cubic and columnar epithelium with few atypical changes in the nucleus. The epithelial cells contain mucin. Furthermore, we observed spindle‐shaped cell proliferation without atypical changes or nuclear division in the stroma. (a) low‐magnification image and (b) high‐magnification image. (c, d) Immunohistochemistry showing that the spindle cells are positive for the estrogen receptor (ER) and progesterone receptor (PgR). The cystic lesion with ovarian‐like stroma was diagnosed as a mucinous cystic neoplasm with low‐grade intraepithelial neoplasia.

We subsequently performed a peroral digital cholangioscopy (SpyScope DSII; Boston Scientific) for the differential diagnosis of the lesions and targeted biopsies (Figure 2). Cholangioscopy revealed translucent oval masses with capillary vessels attached to the bile duct walls. The surface was mostly smooth yet partially irregular with redness (Figure 2a,b), suggesting that the masses were epithelial neoplasms. The masses were attached to the bile duct wall (Figure 2c). Cholangioscopy‐guided targeted biopsy using dedicated biopsy forceps (SpyBite Max; Boston Scientific) revealed a small hole in the lesion (Figure 2d). Finally, we deployed two plastic stents for bilateral drainage considering the tumor extension to the perihilar bile duct. Histological examination of the biopsy specimens revealed subepithelial spindle‐shaped cell proliferation with no atypical epithelial changes. Immunohistochemistry showed that these cells were positive for alpha‐smooth muscle actin, caldesmon, calponin, and HHF35; on the other hand, they were negative for desmin, MyoD1, myogenin, and CK AE1/AE3. These pathological findings suggested the possibility of myogenic tumors, such as rhabdomyosarcoma; however, a definitive diagnosis was difficult because of insufficient and degenerated biopsy materials. The patient underwent extrahepatic bile duct resection to provide treatment based on the accurate pathological diagnosis. Macroscopic examination of the resected specimen revealed a multicystic lesion with a diameter of 30 mm, filled with colorless and transparent mucin. The cystic lesions did not contain multiple nodules. Histopathological evaluation revealed a layer of cubical and columnar epithelium with a few atypical changes in the nucleus (Figure 3a,b). The epithelial cells contained mucin. Furthermore, we observed spindle‐shaped cell proliferation without atypical changes or nuclear division in the stroma. In addition to the findings from the biopsy specimen, immunohistochemistry showed that the spindle cells were positive for the estrogen receptor (ER) and progesterone receptor (PgR) (Figure 3c,d).

Finally, the cystic lesion with ovarian‐like stroma showing positive for ER and PgR was diagnosed as an MCN with low‐grade intraepithelial neoplasia. The horizontal margin of the bile duct was negative for atypical epithelium, and lymph node metastasis was negative. After complete tumor resection, contrast‐enhanced computed tomography was performed every 4 months. No recurrence was observed.

## DISCUSSION

Herein, we report a case of MCN originating from an extrahepatic bile duct. Peroral cholangioscopy revealed translucent oval‐shaped masses with partially irregular surfaces and capillary vessels attached to the bile duct wall. To the best of our knowledge, this is the first report of cholangioscopic findings of biliary MCN.

According to the revised 2019 World Health Organization classification of tumors of the digestive system and Fukuoka guidelines, MCNs are defined as cystic tumors composed of two distinct histological components: an inner epithelial layer composed of tall mucin‐secreting cells and a dense cellular ovarian‐like stroma.^3^, ^4^ MCNs usually occur in middle‐aged women, and patients with MCN are recommended to undergo surgical treatment due to their potential for malignant transformation. However, it is difficult to confirm the diagnosis preoperatively because of the rarity of biliary MCN and the difficulties in lesion biopsy.

We searched previous resected cases of MCN originating from the extrahepatic bile duct, or MCN of the liver growing into the bile duct. In addition to Fukui's report ^5^ and our case, we reviewed six reports of MCN originating from the extrahepatic bile duct (Table 1). The median age was 40 years (range, 25–69 years), and all patients were female. The median tumor size was 55mm (range, 18–135mm). Seventeen out of a total of 26 patients had obstructive jaundice. In all cases, the pathological diagnosis was benign mucinous cyst adenoma.

**TABLE 1:** Review of resected cases of mucinous cystic neoplasm (MCN) originated from the extrahepatic bile duct or MCN of the liver grew into the bile duct.

Case	Year	Author	Age	Sex	Symptoms	Tumor size	Primary site of the tumor	Operation	Pathological diagnosis
1	2004	Shima	62	F	Jaundice	41 mm	CBD	Bile duct resection	Cystadenoma of the CBD
2	2004	Park	42	F	Jaundice	N/A	CBD	Bile duct resection	Biliary cystadenoma
3	2004	Preetha	58	F	Hypochondrial pain, jaundice	N/A	LHD, CBD	Left hemihepatectomy	Biliary cystadenoma
4	2006	Baudin	40	F	Epigastric pain and jaundice	70 mm	Left lobe	Left hemihepatectomy	Biliary cystadenoma
5	2009	Gonzalez	32	F	Abdominal pain and jaundice	79 mm	S3	Left hemihepatectomy	Biliary cystadenoma
6	2009	Siriwardana	25	F	Hypochondrial pain	55 mm	S4	Left hemihepatectomy and cholecystectomy	Biliary cystadenoma
7	2009	Yi	56	F	Hypochondrial pain and jaundice	55 mm	S4	Left hemihepatectomy	Biliary cystadenoma
8	2010	Saravanan	34	F	Jaundice	45 mm	S4	Bile duct resection	Biliary cystadenoma
9	2011	Hennessey	54	F	Abdominal pain	18 mm	CBD	Bile duct resection	Biliary cystadenoma
10	2011	Harmouch	57	F	Hypochondrial pain and jaundice	50 mm	S4	Left hemihepatectomy, bile duct resection, and cholecystectomy	Hepatobiliary cystadenoma
11	2012	Vyas	41	F	Epigastric pain	30 mm	S4	Left hemihepatectomy and bile duct resection	Hepatobiliary cystadenoma
12	2012	Soochan	62	F	Dysuria	N/A	LHD	Extended left hemihepatectomy and bile duct resection	Extrahepatic cystadenoma
13	2012	Abe	28	F	Abdominal pain	73 mm	S4	Segmentectomy, bile duct resection, and cholecystectomy	Hepatobiliary cystadenoma
14	2013	Rayapudi	37	F	Abdominal bloating	29 mm	S4	Left hemihepatectomy and bile duct resection	Biliary cystadenoma
15	2013	Chandrasinghe	39	F	Jaundice	N/A	S4	Left hemihepatectomy and bile duct resection	Biliary mucinous cystadenoma
16	2015	Takano	57	F	Abdominal pain and fever	83 mm	S4	Left hemihepatectomy, bile duct resection, and cholecystectomy	MCN‐L
17	2015	Takano	26	F	Jaundice	61 mm	S4	Extended left hemihepatectomy and bile duct resection	MCN‐L
18	2018	Pattarapuntakul	27	F	Jaundice	56 mm	CBD	Left hemihepatectomy	MCN‐L
19	2021	Fukui	69	F	Hypochondrial pain and jaundice	40 mm	S4	Left hemihepatectomy and bile duct resection	MCN‐L
20	2010	Sukanta	55	F	Epigastric pain, jaundice, and fever	100 mm	porta hepatis	Bile duct resection	Biliary mucinous cystadenoma
21	2020	Aljubran	48	F	Jaundice	20 mm	CBD	Bile duct resection	Biliary mucinous cystadenoma
22	2020	Srinivas	31	F	Abdominal pain, vomiting, fever, and obstructive jaundice	47 mm	S4	Left hemi‐hepatectomy	MCN‐L and Biliary mucinous cystadenoma
23	2021	Paspala	55	F	Abdominal pain, jaundice	20 mm	CBD	PPPD	Biliary mucinous cystadenoma
24	2022	Chen	28	F	Abdominal pain	70 mm	LHD	Bile duct resection	Biliary mucinous cystadenoma
25	2022	Dhali	26	F	Epigastric pain	135 mm	S4+5	Enucleation of the mass	Biliary mucinous cystadenoma
26	2023	Present case	29	F	Jaundice	30 mm	Common hepatic duct	Bile duct resection	Biliary mucinous cystadenoma

Abbreviations: CBD, common bile duct; F, female; LHD, left hepatic duct; MCN‐L, mucinous cystic neoplasm of the liver; N/A, not applicable; PPPD, pylorus preserving pancreatoduodenectomy; S, segment.

In the present case, CT, MRI, and EUS suggested that the lesion mainly consisted of a cystic component with a thin external capsule, and the endoscopic retrograde cholangiography showed round contrast defects, which is consistent with previous reports.^6^, ^7^, ^8^ Intraductal papillary neoplasm of the bile duct (IPNB) is also a cystic tumor that produces mucin; thus, it is often difficult to distinguish biliary MCN from IPNB using these imaging modalities.^1^ In our case, cholangioscopic imaging revealed translucent oval‐shaped masses with smooth and mostly normal epithelia, suggesting that the masses were unlikely to be IPNB. These cholangioscopic findings reflect the histopathological findings, in which the masses were composed of a layer of cubical and columnar epithelium with few atypical changes in the nucleus and contained colorless and transparent mucin in the cavity. Thus, cholangioscopic imaging was helpful in confirming the preoperative diagnosis.

In the hepatobiliary tract, almost all MCNs have been reported to arise from the liver, and reports on extrahepatic MCNs are extremely rare.^9^ Although most resected hepatobiliary MCNs are reported to be benign,^1^ complete surgical resection of the tumor is needed because of its malignant potential and risk of recurrence. In the present case, we identified the demarcation line of the tumor by directly viewing the protrusion on the bile duct surface and performing a targeted step biopsy under cholangioscopy. This detailed preoperative endoscopic examination contributed to the complete surgical removal of the tumor. Therefore, extrahepatic bile duct resection was performed for complete surgical removal. Cholangioscopic imaging can help determine the operative form and resection line.

In conclusion, we report a case of resected MCN originating from the extrahepatic bile duct. Cholangioscopic imaging can be helpful in the differential diagnosis of biliary neoplasms and in the determination of treatment strategies.

## CONFLICT OF INTEREST STATEMENT

None.
